# Supplementary material for: Experiences of immigrants when accessing mental health services and psychosocial supports in Canada: Protocol for a scoping review
Source: PLoS One. 2025 Apr 15;20(4):e0319743. doi: 10.1371/journal.pone.0319743 (PMC11999126; doi:10.1371/journal.pone.0319743)
Supplement: S2 Table — (DOCX) [file pone.0319743.s002.docx]

S2_Supporting Information Draft Strategy for Medline

| Study Characteristics | Details |
| --- | --- |
| Author(s) | List all authors' last names and initials |
| Publication Year | Year the study was published |
| Study Title | Full title of the study |
| Journal Name | Name of the journal where the study was published |
| Study Design | e.g., qualitative, quantitative, mixed-methods, etc. |
| Study Objectives | Brief summary of the study's main objectives or research questions |
| Province/Territory | Specific province or territory within Canada where the study was conducted |
| Notes |  |
| Relevant articles from the reference list |  |
| Participant Characteristics | |
| Sample Size | Total number of study participants |
| Age Range | Age range of the study participants |
| Gender | Number/percentage of male, female, and other participants |
| Ethnicity/Cultural Background | Specific ethnic or cultural groups represented in the study sample |
| Immigration Status | e.g., recent immigrants, refugees, asylum seekers, etc. |
| Length of Time in Canada | Range or average length of time participants have been in Canada |
| Language Proficiency | Participants' proficiency in English, French, or other languages |
| Education Level | Participants' highest level of education |
| Mental Health Conditions | Specific mental health conditions experienced by participants (e.g., depression, anxiety, PTSD) |
| Comorbidities | Presence of other health conditions in addition to mental health issues |
| Service Access and Utilization | |
| Types of Services Accessed | e.g., counseling, therapy, support groups, medication management, etc. |
| Setting of Services | e.g., community health center, hospital, private practice, etc. |
| Frequency of Service Use | How often participants accessed mental health services |
| Duration of Service Use | Length of time participants used mental health services |
| Key Findings | |
| Experiences with Services | Summary of participants' positive and negative experiences with mental health services |
| Barriers to Access | Specific barriers that prevented or hindered participants from accessing services |
| Enablers of Access | Factors that facilitated or enabled participants' access to services |
| Recommendations | Recommendations made by the study authors for improving access and experiences |
