# Supplementary material for: Experiences of immigrants when accessing mental health services and psychosocial supports in Canada: Protocol for a scoping review
Source: PLoS One. 2025 Apr 15;20(4):e0319743. doi: 10.1371/journal.pone.0319743 (PMC11999126; doi:10.1371/journal.pone.0319743)
Supplement: S3 File — (DOCX) [file pone.0319743.s003.docx]

**Search Strategy**

Databases:

1. MEDLINE (Ovid)
2. Embase (Ovid)
3. PsycINFO (Ovid)
4. CINAHL (EBSCOhost)
5. Social Work Abstracts (EBSCOhost)
6. SocINDEX (EBSCOhost)
7. Scopus
8. Web of Science Core Collection

Search Terms:

1. Immigrant-related terms: immigrant* OR migrant* OR refugee* OR newcomer* OR "asylum seeker*" OR "foreign born" OR "non-native" OR "foreign national*" OR "naturalized citizen*"
2. Mental health-related terms: "mental health" OR "mental illness*" OR "mental disorder*" OR "psychiatric disorder*" OR "psychological distress" OR depression OR anxiety OR trauma OR "post-traumatic stress disorder" OR PTSD OR "substance use disorder*" OR addiction
3. Service access-related terms: "health services accessibility" OR access* OR utilization OR "health services use" OR "service use" OR barrier* OR facilitator* OR enabler* OR "unmet need"
4. Psychosocial support-related terms: "social support" OR "psychosocial support" OR "community support" OR "peer support" OR "family support"
5. Canada-related terms: Canada OR "Newfoundland and Labrador" OR "Prince Edward Island" OR "Nova Scotia" OR "New Brunswick" OR Quebec OR Ontario OR Manitoba OR Saskatchewan OR Alberta OR "British Columbia" OR Yukon OR "Northwest Territories" OR Nunavut
6. Combine the search terms with Boolean operators: (1 AND (2 OR 3 OR 4) AND 5)

Limits:

- Publication date: 2014-2024
- Language: English
- Publication type: peer-reviewed journal articles
- Study design: quantitative, qualitative, or mixed-methods studies

Search String Example (Ovid MEDLINE):

((immigrant* OR migrant* OR refugee* OR newcomer* OR "asylum seeker*" OR "foreign born" OR "non-native" OR "foreign national*" OR "naturalized citizen*") AND ("mental health" OR "mental illness*" OR "mental disorder*" OR "psychiatric disorder*" OR "psychological distress" OR depression OR anxiety OR trauma OR "post-traumatic stress disorder" OR PTSD OR "substance use disorder*" OR addiction OR "health services accessibility" OR access* OR utilization OR "health services use" OR "service use" OR barrier* OR facilitator* OR enabler* OR "unmet need" OR "social support" OR "psychosocial support" OR "community support" OR "peer support" OR "family support") AND (Canada OR "Newfoundland and Labrador" OR "Prince Edward Island" OR "Nova Scotia" OR "New Brunswick" OR Quebec OR Ontario OR Manitoba OR Saskatchewan OR Alberta OR "British Columbia" OR Yukon OR "Northwest Territories" OR Nunavut))
